# An Imbalanced Fault Diagnosis Method Based on Multi-Sensor Selection and Graph Attention Mechanism

**DOI:** 10.3390/s26041182

**Published:** 2026-02-11

**Authors:** Qiangqiang Xiong, Qiming Shu, Ke Wu, Jun Wu, Jianwen Hu

**Affiliations:** 1 Jiangxi Key Laboratory of Modern Agricultural Equipment Jiangxi Province, College of Engineering, Jiangxi Agricultural University, Nanchang 330045, China; xqqwok@jxau.edu.cn; 2 School of Naval Architecture and Ocean Engineering, Huazhong University of Science and Technology, Wuhan 430074, China; 3 Hubei ChuTianYun Co., Ltd., Wuhan 430070, China; kewu@chutianyun.gov.cn; 4 Institute for Math & AI (IMAI), Wuhan University, Wuhan 430072, China; 5 School of Software, Jiangxi Agricultural University, Nanchang 330045, China; jianwen.hu@jxau.edu.cn

**Keywords:** fault diagnosis, graph attention network, imbalanced dataset, bearing

## Abstract

Severe diagnostic errors are often caused by the significant imbalance between normal and fault data in bearing datasets. To solve this challenge, a graph attention convolutional neural network based on sensitivity analysis and correlation analysis (SCGAT) is proposed to achieve bearing fault diagnosis under imbalanced-dataset conditions. Firstly, a graph attention convolutional neural network is constructed to effectively extract fault-related features from multi-sensor data. Then, a sensor sensitivity analysis module is built to filter and select effective sensor information. A sensor correlation analysis module is introduced to distinguish the correlation between different sensors, and strongly correlated sensors are merged. Finally, the merged features are input into a classifier for fault diagnosis. The effectiveness of the proposed method is verified on a power transmission simulation experiment platform. The experimental results show that the proposed SCGAT can effectively achieve fault diagnosis under imbalanced data conditions, and exhibits higher diagnostic accuracy and stability compared to other models.

## 1. Introduction

Bearings are regarded as a kind of typical rotating machinery and are widely used in various power transmission equipment [1,2]. The equipment is characterized by large size, complex structure and heavy load. Almost all torque is transmitted through these components. Once a fault occurs, it will cause the connected components to operate abnormally, and ultimately lead to a bearing fault. This poses a huge safety hazard to power equipment [3,4,5,6,7]. Consequently, it is important to carry out fault diagnosis for the stable and safe operation of bearings.

Typical fault diagnosis methods include oil analysis [8], acoustic emission [9], temperature detection, and vibration detection. All these methods monitor data through sensors and then analyze the working state of the equipment. However, fault diagnosis techniques have been improved significantly with the advancement of sensor technology and artificial intelligence technology. Artificial intelligence technology is used to extract the time–frequency domain feature from vibration signals and construct a fault diagnosis model to determine the fault type. It is the mainstream method for bearing fault diagnosis [10,11,12].

Statistics-based artificial intelligence methods are employed to process data and extract features. These include frequency domain analysis methods, time domain analysis methods, and time–frequency domain analysis methods. The frequency domain analysis methods mainly use the Fourier transform to obtain the frequency domain features of the time domain signal, and then achieve fault diagnosis by analyzing the frequencies of different vibration signals [13,14]. The time domain analysis methods directly analyze the signal features collected by the sensor without processing [15,16]. The time–frequency domain analysis methods can not only analyze the relationship between the time and frequency of the vibration signal but also extract the local detail features of the signal, and they are capable of processing non-stationary and non-linear signals [17,18,19]. However, these methods all require manual extraction of the fault features. This means relying on the extensive experience of professionals. Due to the limitations in the applicability of these methods, they cannot meet the requirements of long-term safe and stable operation of bearings.

Deep learning techniques have become an effective method for bearing fault diagnosis. For example, Xie et al. proposed a feature-highlighting processing method based on a reverse-attention mechanism to highlight important features [20]. Gao et al. used the mini-batch stochastic gradient descent method to pre-train the DBN, and then used the backpropagation neural network and conjugate gradient descent method to supervise and fine-tune the entire DBN model, effectively improving the fault diagnosis accuracy of the model [21]. Zhang et al. proposed a multi-scale, full-spectrum CNN model to address the problem of poor fault diagnosis performance under variable working conditions, which ensures classification ability while avoiding overfitting caused by overly complex networks [22]. Yuan et al. extracted temporal feature information through the bidirectional long short-term memory network and performed fault classification in CatBoost, the experiments showed that the fault diagnosis accuracy was high [23].

Deep learning requires a large amount of data. Among the data collected by numerous sensors, the data from a few sensors are merely noise information. Therefore, addressing the noise problem caused by having multiple sensors has also led to many research achievements [24,25,26,27]. Wang et al. presented an improvement of a convolutional autoencoder network to merge and exploit the valid features generated from multi-sensor data [28]. There are also other methods for fault diagnosis when using multiple sensors to monitor the working condition of bearings [29,30,31,32,33,34,35,36]. Kim et al. developed a new potentially linear model that builds HI and selects an informative sensor to depict the regression course in a harmonized way [37]. Melluso et al. proposed a non-invasive torque FD framework. The residual is refined through a Recursive Least Squares estimator to enhance the adaptability of the proposed modeling approach [38]. Wang et al. proposed an intelligent process fault diagnosis system through integrating the techniques of an Andrews plot and convolutional neural networks [39]. Wang et al. proposed a new fault diagnosis method based on the distance and probability topological graph (DPGCN) model to address the common problem of imbalanced classes in marine diesel-engine condition monitoring data, which can improve the classification accuracy and stability under imbalanced data sets [40]. Zhang et al. proposed a braking friction fault diagnosis method based on a one-dimensional convolutional neural network (1DCNN) and GraphSAGE network to address the problem of imbalanced fault samples in actual high-speed train braking friction operation. The effectiveness of the network was verified with normal samples much more than the fault samples [41].

The existing diagnosis methods require sufficient fault data. However, fault data is much less plentiful than normal data in practical scenarios. This imbalance in normal data and fault data has raised the difficulty in fault diagnosis.

There are two ways to extract a feature from low-occurrence fault data effectively. The first way is to find an appropriate method to extract the feature effectively. The graph construction method takes samples as nodes and constructs the relationship between each node, and a graph network is generated. The features of the low-occurrence samples can be extracted by the relationships between the low-occurrence samples and the nodes, as well as the features of the neighboring samples, so the GCN and GAT are potential methods. The other way is to find a method to decrease the unwanted signal; this can decrease the difficulty of feature extraction. The sensor selection algorithm is a potential method. A novel graph attention convolutional neural network based on sensitivity analysis and correlation analysis (SCGAT) is proposed. The main contributions of this paper are as follows:

(1) A graph attention convolutional neural network is constructed to effectively extract imbalanced features. This method can capture hidden fault-related features within the dynamic graph structure.

(2) A sensor sensitivity analysis module is built to select sensors highly sensitive to faults. Furthermore, a sensor correlation analysis module is introduced to reveal the strong correlation between sensors.

(3) Experiments are conducted to analyze the performance of the proposed method. This demonstrated that the proposed method can enhance diagnosis accuracy under imbalanced-dataset conditions.

The rest of this paper is organized as follows. In Section 2, we describe the network structure of SCGAT. In Section 3, a power transmission simulation experiment platform is introduced, and the effectiveness of the proposed method is verified. Section 4 gives the conclusions and looks forward to future work.

## 2. Materials and Methods

The proposed SCGAT network model integrates the graph attention network, which is capable of handling dynamic graphs, and a convolutional neural network, which has the advantage of efficient feature extraction. By doing so, it can process the constructed dynamic graphs and extract the features of fault samples efficiently. Each node in the dynamic graph represents a sample, and the connections between nodes represent the relationships between the samples. The samples are represented using the sensor group selected through sensitivity analysis and correlation analysis. The number of sample features is consistent with the number of sensors.

**Figure 1:**
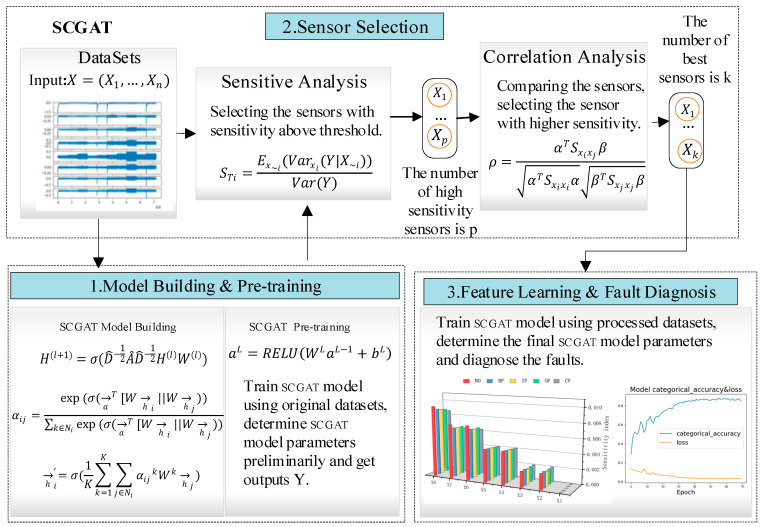
SCGAT network flowchart.

**Figure 2:**
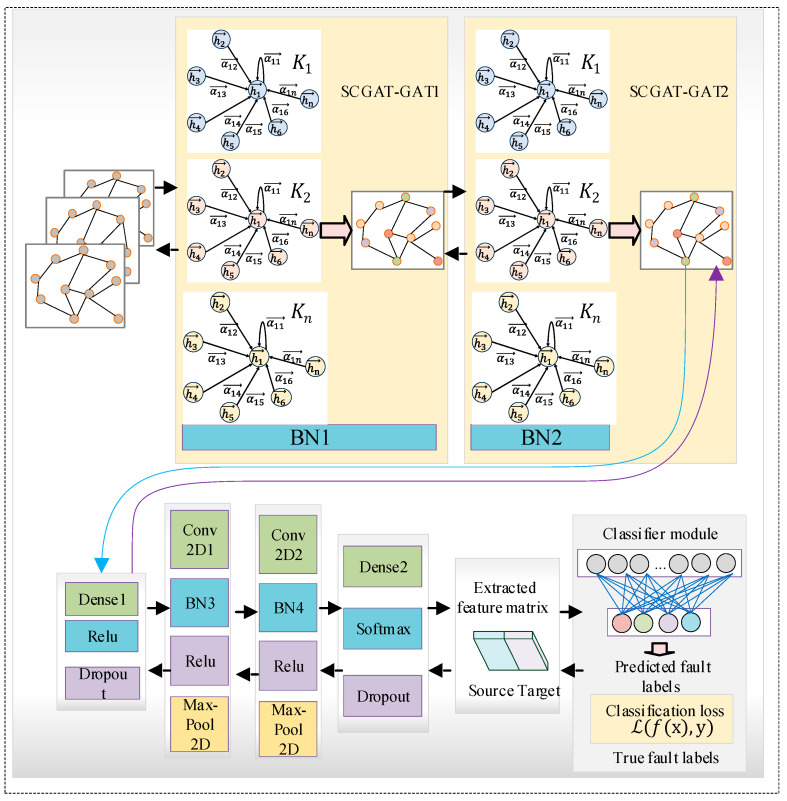
Graph attention convolutional neural network structure.

The SCGAT network flow and structure are shown in Figure 1 and Figure 2. The SCGAT method consists of three parts: (1) the model-building and pre-training stage; (2) the sensor selection stage; (3) the feature-learning and fault diagnosis stage.

The SCGAT diagnosis model is constructed in the model-building and pre-training stage. The complete dataset without sensor screening is used as the input. The hyperparameters of the model are initially determined, and the output of the model is obtained. In the sensor selection stage, sensitivity analysis is employed for sensor screening and selection. Combined with correlation analysis, the optimal sensor group is obtained. In the feature-learning and fault diagnosis stage, the optimal sensor data is used to train the model. The trained model is employed for fault diagnosis, and the diagnostic results are obtained.

### 2.1. Graph Attention Convolutional Neural Network

The graph attention convolutional neural network [42] comprises three components, namely the graph data construction module, the graph attention mechanism module and the convolutional neural network module. Extracting the features of the low-occurrence samples is difficult. The graph construction method takes the samples as nodes and constructs the relationship between each node, and the graph network is generated. The features of the low-occurrence samples can be extracted by the relationships between the low-occurrence samples and the nodes, as well as the features of the neighboring samples, which improves the efficiency of feature learning.

(1) Graph data construction module

The elements in the adjacency matrix are the relationships between each pair of nodes. The relationships between nodes and nodes constitute a dynamic graph. Constructing the adjacency matrix involves calculating the relationship values between each pair of nodes. There are various methods, such as the Euclidean-distance method, cosine similarity method, Pearson correlation coefficient, Spearman correlation coefficient, and Kendall correlation coefficient. The cosine similarity method was selected in this paper after comparative analysis. Nodes can be regarded as samples with features. The new feature of a node is updated by combining the node’s own feature with the relationships between the node and all other nodes.

Let $G=\left(V,E\right)$ be an undirected graph, where the vertex set $V=v_{1},\ldots ,v_{n}$ and the edge set $E=e_{1},\ldots ,e_{\varepsilon }$. Let $a_{ij}$ represent the number of edges between vertices $v_{i}$ and $v_{j}$, and the resulting matrix $A=A(G)={\left(a_{ij}\right)}_{n\times n}$ is the adjacency matrix of graph G. A propagation rule is given: f(hⁱ,A)=relu(AhⁱWⁱ), where $h^{i}$ is the feature of the i-th layer, $W^{i}$ is the weight matrix of the i-th layer, and relu is a non-linear activation function. Assume the initial conditions: (1) i = 1 and f acts on the input feature matrix. (2) relu is the identity function. (3) Select the weights, $Ah^{0}W^{0}=AXW^{0}=AX$; that is, f(X,A)=AX, where $h^{0}$ is the feature of the input layer, and $W^{0}$ is the weight of the input layer. The representation of each node is the sum of the features of its adjacent nodes, that is, the aggregation of adjacent nodes.

Since the aggregated representation of nodes is the feature aggregation of adjacent nodes, their own features not included, a self-loop is added to each node before applying the propagation rule; that is, the adjacency matrix A is added to the identity matrix I. The connection situation of nodes is described by the degree matrix, that is, the number of nodes including itself. Nodes with large degrees have larger values in their feature representations, while nodes with small degrees have smaller values. This can lead to gradient vanishing or explosion and affect the stochastic gradient descent algorithm. Therefore, by multiplying the adjacency matrix A with the inverse of the degree matrix D, a transformation is performed to normalize the feature representations by the degree of the nodes. The simplified propagation rule is $f(X,\hat{A})={\hat{D}}^{-\frac{1}{2}}\hat{A}{\hat{D}}^{-\frac{1}{2}}X$, where $\hat{D}$ is the degree matrix corresponding to $\hat{A}=A+I$, the degree matrix of the matrix A with forced self-loops; then, applying the relu activation function, it becomes a complete layer with the adjacency matrix, input feature, weight, and activation function.

(1) $$f(X,\hat{A})=relu({\hat{D}}^{-\frac{1}{2}}\hat{A}{\hat{D}}^{-\frac{1}{2}}XW)$$

The expression can be expressed as the following formula for any layer:

(2) $$h^{\left(l+1\right)}=relu({\hat{D}}^{-\frac{1}{2}}\hat{A}{\hat{D}}^{-\frac{1}{2}}h^{\left(l\right)}W^{\left(l\right)})$$

(2) Graph attention mechanism module

A node has multiple neighboring nodes, and the importance of these neighboring nodes to a particular node varies a lot. Therefore, an attention mechanism is introduced to calculate the attention coefficients between the node and the neighboring nodes, and to weight and sum the features of the neighboring nodes; this makes the feature update of the node more reasonable.

The graph attention mechanism calculates the attention coefficients between nodes and other nodes first and normalizes them, then performs weighted summation. If multiple attention coefficients are calculated simultaneously, the results of the K attention mechanisms are horizontally concatenated or averaged. The input and output of each layer are as follows:

1. Input: $h\;=\;\text{\{}\overset{⃑}{h_{1}},\overset{⃑}{\;h_{2},}\text{…},\;\overset{⃑}{h_{N},}\text{\}},\;\overset{⃑}{h_{i}}\in R^{F}$
2. Output: $h^{\prime }\;=\;\text{\{}\overset{⃑}{{h^{\prime }}_{1}},\overset{⃑}{{{\;h}^{\prime }}_{2},}\text{…},\overset{⃑}{{{\;h}^{\prime }}_{N},}\text{\}},\overset{⃑}{{{\;h}^{\prime }}_{i}}\in R^{F}$

The formula for calculating the attention coefficient between node i and node j:

(3) $$\alpha_{ij}=softmax(LeakyReLU({\overset{⃑}{a}}^{T}[W\overset{⃑}{h_{i}}||W\overset{⃑}{h_{j}}]))$$

Firstly, calculate the similarity between vertex i and its neighboring nodes $j\in N_{i}$:

(4) $$e_{ij}=a(W\overset{⃑}{h_{i}},W\overset{⃑}{h_{j}})$$

In the formula, $(W\overset{⃑}{h_{i}},W\overset{⃑}{h_{j}}$) indicates that the features $h_{i}$ and $h_{j}$ share the parameter matrix W, and both use W to perform linear transformation on the features. In the formula represents horizontal concatenation, and thI outermost a in the formula represents a single-layer feedforward neural network, using LeakyReLU as the activation function, and the output is a numerical value. With the correlation coefficient, it is then normalized using Softmax.

(5) $$\alpha_{ij}=\frac{exp(e_{ij})}{\sum_{k\in N_{i}}exp(e_{ik})}$$

(6) $$\alpha_{ij}=\frac{exp(LeakyReLU({\underset{a}{\to }}^{T}[W{\underset{h}{\to }}_{i}||W{\underset{h}{\to }}_{j}]))}{\sum_{k\in N_{i}}exp(LeakyReLU({\underset{a}{\to }}^{T}[W{\underset{h}{\to }}_{i}||W{\underset{h}{\to }}_{j}]))}$$

**Figure 3:**
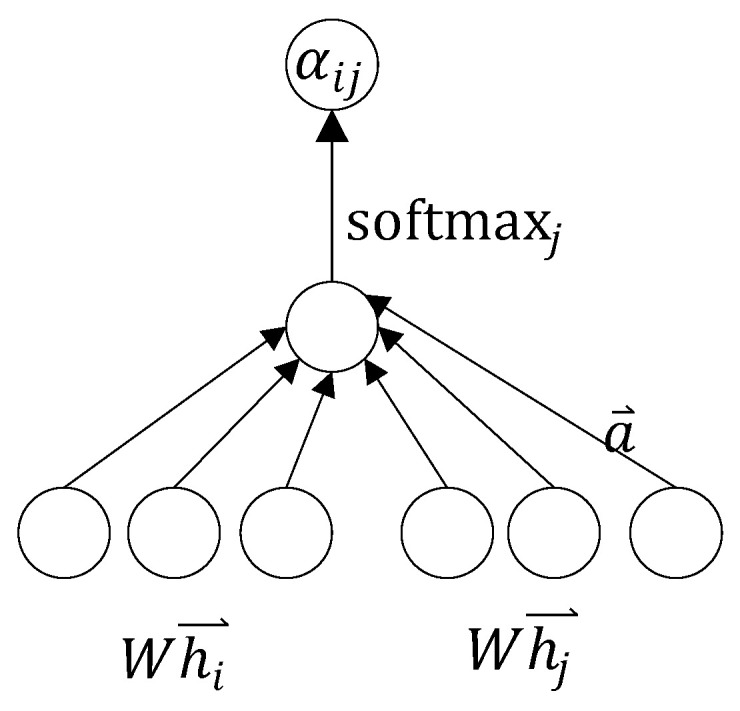
Calculation process of attention coefficient.

**Figure 4:**
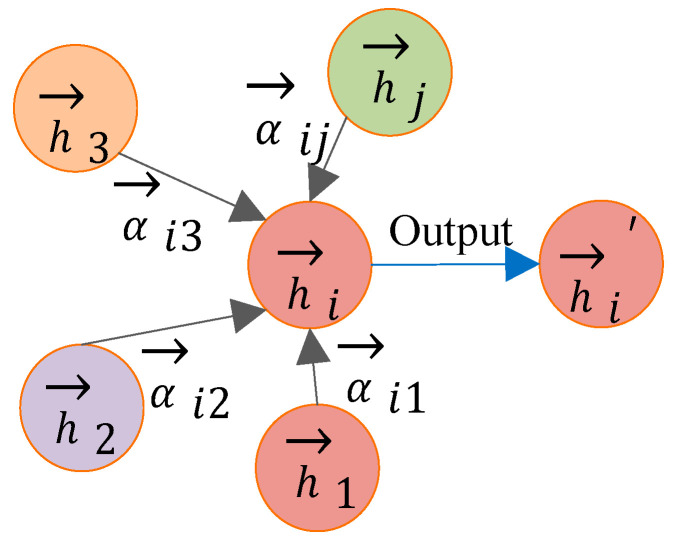
The concatenation process of a single attention mechanism.

Here, || represents vector concatenation. The calculation process of the attention coefficient is shown in Figure 3:

After obtaining the attention coefficient, the features of the neighboring nodes are weighted and summed. The concatenation process of a single attention mechanism is shown in Figure 4:

(7) $${\underset{h}{\to }}_{i}^{\prime }=relu(\sum_{j\in N_{i}}\alpha_{ij}W{\underset{h}{\to }}_{j})$$

**Figure 5:**
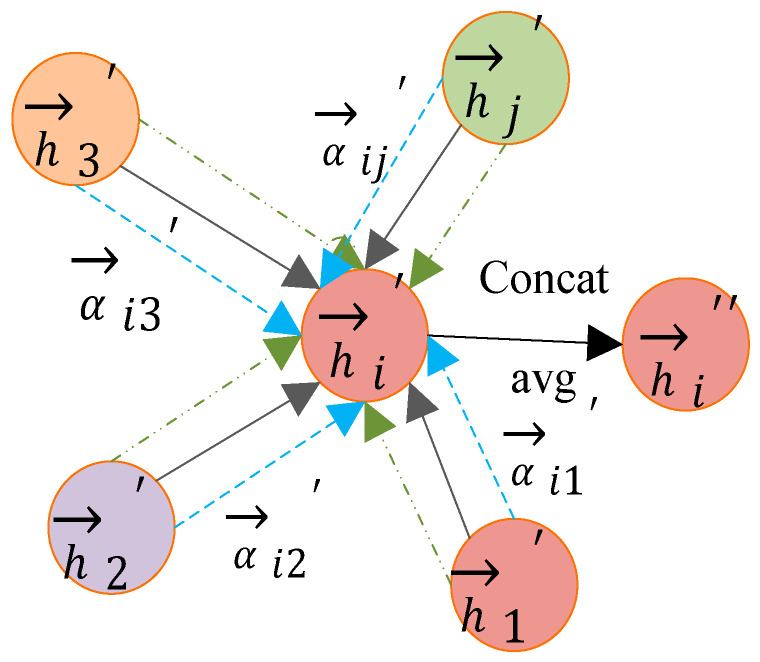
The concatenation process of multiple attention mechanisms.

For better learning results, multiple attentions are used. For K attentions, two methods can be employed to aggregate the neighboring nodes. One method is the horizontal concatenation approach, where the aggregated-feature dimension is K times the original one. The other method is to take the average of the results obtained from the K attention mechanisms. The concatenation process of multiple attention mechanisms is shown in Figure 5.

(8) $${\underset{h}{\to }}_{i}^{\prime }={\left|\right|}_{k=1}^{K}relu(\sum_{j\in N_{i}}{\alpha_{ij}}^{k}W^{k}{\underset{h}{\to }}_{j})$$

(9) $${\underset{h}{\to }}_{i}^{\prime }=relu(\frac{1}{K}\sum_{k=1}^{K}\sum_{j\in N_{i}}{\alpha_{ij}}^{k}W^{k}{\underset{h}{\to }}_{j})$$

(3) Convolutional neural network module

Although the graph attention network can handle dynamic graphs, the neighbors extracted for each vertex are different, which makes the calculation processing necessary for each vertex. The effectiveness of the feature extraction is not as good as that of a convolutional neural network. Therefore, a convolutional neural network is integrated into the SCGAT method to improve the effect of feature extraction.

The modules of the convolutional neural network mainly include the convolution module and the pooling module. The convolution module includes expanding boundaries, convolution windows, forward convolution, backward convolution, etc., and the pooling module includes forward pooling, creating masks, value allocation, backward pooling, etc. A network with convolution layers and pooling layers contains forward and backward propagation. During the forward-propagation process, some values are stored to calculate the gradient values in the backward-propagation process. Using the output of the graph attention network module as the input of the convolutional neural network module, features are extracted from the dynamic graph, and the extracted features are classified using a fault classifier to achieve fault diagnosis of bearings under the condition of an imbalanced dataset.

### 2.2. Sensitivity Analysis of Sensor

Due to the large number of sensors used for monitoring, there are differences in the diagnostic effects of different sensors. Sensors with poor diagnostic performance can be used for fault diagnosis, resulting in a large amount of interference information carried by a single sample, making it difficult to diagnose. Therefore, sensitivity analysis and correlation analysis are used to screen the sensors with many interference factors to improve the accuracy of fault diagnosis.

Sensitivity analysis is a type of uncertainty analysis technique that studies the degree to which changes in certain factors affect one or more key indicators from a quantitative perspective. The sensitivity of each input is represented by a sensitivity index. The sensitivity index includes the following types: the first-order index measures the contribution of a single model input to the variance of the output; the second-order index measures the contribution of the interaction between two model inputs to the variance of the output; and the total-order index measures the contribution of model inputs to the variance of the output, including the indexes of first-order and higher-order [43]. Generally, the sensitivity index can be divided into four grades, as shown in Table 1.

The average sensitivity index refers to the average value of the total sensitivity index of this characteristic parameter across several types of faults, where r represents the total number of fault categories.

(10) $$mean\left(S_{Tj}\right)=\frac{{S_{Tj}^{1}+S_{Tj}^{2}\ldots +S}_{Tj}^{r}}{r}$$

The model is set as y = f(x), where $x=x_{1},{\;x}_{2},\ldots ,\;x_{N}$ are the input values of the model; $x_{j}$ represents the j-th column of data, which is a column vector; N indicates the number of features, and it follows a uniform distribution over [0,1]; and $f^{2}(x)$ is integrable. y is the corresponding output value. The model can be decomposed into the sum of functions of different dimensions:

(11) $$f_{0}=\int f\left(x\right)dx$$

(12) $$f\left(x\right)=f_{0}+\sum_{s=1}^{N}\sum_{j_{1}<\ldots <j_{s}}^{N}f_{j_{1}\ldots j_{s}}(x_{j_{1}},\ldots ,{\;x}_{j_{s}})$$

Here, $1<j_{1}<\ldots <j_{s}<N,\;\;f_{j_{1}\ldots j_{s}}$ represents the functional relationship between the j-th feature parameter and the other feature parameters; the total variance v of the model can be calculated using the following formula:

(13) $$v=\int f^{2}dx-{f_{0}}^{2}$$

(14) $$v_{j_{1}\ldots j_{N}}=\int f_{j_{1}\ldots j_{N}}^{1}dx_{j_{1}}\ldots dx_{j_{N}}$$

The overall variance of the model can also be decomposed into the influence of individual feature parameters and the combined effect of each feature parameter:

(15) $$v=\sum_{j=1}^{N}\sum_{j_{1}<\ldots <j_{N}}^{N}v_{j_{1}\ldots j_{N}}$$

In the formula, v represents the total variance of the model output y. $v_{j_{1}\ldots j_{N}}$ represents the variance term of the j-th characteristic parameter acting together with all other characteristic parameters. By normalizing the above equation, the total sensitivity index $S_{Tj}$ of the j-th characteristic parameter can be obtained, which is used to represent the total influence of the j-th characteristic parameter on the model and the mutual influence among the characteristic parameters.

(16) $$S_{Tj}=S_{j_{1}\ldots j_{N}}=\frac{v_{j_{1}\ldots j_{N}}}{v}$$

The above calculation process is based on the model y = f(x), and further extends the model to SCGAT. During pre-training and pre-diagnosis, a set of data was calculated. The original data X was used as the input x, and the diagnostic result Y was used as the output y. The calculation of the sensitivity index is the same as the above process.

Sensitivity analysis assesses the influence of parameters on the response, which can be divided into local sensitivity and global sensitivity in the form of partial derivatives. Variance analysis assesses the contribution of input variance to response variance, which is a type of local sensitivity. From the above analysis, it can be known that parameters with larger variance have larger local sensitivity. The statistical significance of variance is that when the data distribution is relatively scattered, that is, when the data fluctuates more around the average value, the sum of the squares of the differences between each data point and the average value is larger, and the variance is larger. When the data distribution is relatively concentrated, the sum of the squares of the differences between each data point and the average value is smaller; so the smaller the variance, the smaller the fluctuation of the data. When selecting the data measured by sensors to train the model and perform fault diagnosis, the smaller the data fluctuation, the more conducive it is to improving the accuracy rate. Therefore, when screening sensors, sensors with sensitivity indices lower than the average value are selected to obtain the sensitive-sensor group, thereby improving the fault diagnosis effect.

### 2.3. Correlation Analysis Among Sensors

Sensors with more interfering information can be eliminated through sensitivity analysis. To improve the fault diagnosis effect, the results of correlation analysis are utilized to merge the sensors with extremely strong correlations. The sensor with the higher average sensitivity index among the two is removed. Finally, the remaining sensors are regarded as the optimal sensor group.

Correlation analysis was conducted using the original data of the sensors, and the correlation between the sensors was calculated. A correlation coefficient of 0.8 or above indicates a strong correlation. The larger the absolute value of the correlation coefficient, the stronger the correlation. The closer the correlation coefficient is to 1 or −1, the stronger the correlation. The closer the correlation coefficient is to 0, the weaker the correlation. Generally, the strength of variable correlation is determined by the value range shown in Table 2.

Suppose two sets of vectors are given: $X={\left(x_{1},x_{2},\ldots ,x_{u}\right)}^{T}$, $Y=({x_{u+1},x_{u+2},\ldots ,x_{u+v})}^{T}.$ Among them, u and v are the dimensions of the two sets of vectors, where u≤v, u+v=M. Select several representative linear variables $P_{i}$ and $Q_{i}$ from X and Y respectively. These linear variables are all linear combinations of the original variables; that is, $P_{i}=\alpha^{T}X,Q_{i}=\beta^{T}Y$. To obtain the maximum correlation between the two sets of variables, calculate their Pearson correlation coefficient $\rho (P_{i,}Q_{i})$.

(17) $$argmax\rho =corr\left(X,Y\right)=\frac{\alpha^{T}S_{xy}\beta }{\sqrt{\alpha^{T}S_{xx}\alpha }\sqrt{\beta^{T}S_{yy}\beta }}$$

In the equation, $\alpha^{T}S_{xx}\alpha =1$, $\beta^{T}S_{yy}\beta =1$, α and β represent the typical correlation between $P_{i}$ and $Q_{i}$ in the projection directions, $S_{xx}$ and $S_{yy}$ are the covariance matrices of vectors X and Y respectively, and $S_{xy}$ is the covariance matrix between X and Y.

The original data of the sensor group obtained from the sensitivity analysis was divided into different columns according to different sensors, all the data of one sensor corresponds to one variable. All the variables are combined in pairs. After obtaining the first set of related variables, the related variables of other groups are calculated. This process continues until the correlation between the variables is extracted completely, that is, until the correlation coefficients between each pair of sensors are determined. For combinations with correlation coefficients greater than the average value, the sensors with lower sensitivity indices are retained; thus, the optimal sensor group is obtained.

## 3. Results

To verify the effectiveness of the proposed SCGAT at the task of fault diagnosis under the condition of an imbalanced dataset, several different methods were introduced and compared with the proposed method. Experimental verification and effect analysis were conducted on the bearing dataset.

### 3.1. Experimental Platform and Dataset Introduction

**Figure 6:**
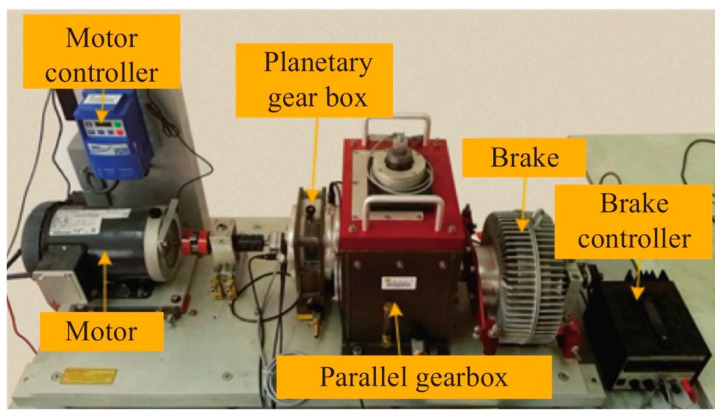
Power transmission simulation experimental platform.

The bearing dataset was collected from the gearbox data of the power transmission simulation experiment platform at Southeast University. This platform mainly consists of a motor, motor controller, gearbox, planetary gearbox, brake, and brake controller, and can simulate various bearing operating conditions, as shown in Figure 6.

This experiment simulated five bearing operating conditions under two working conditions: a rotational speed of 20 Hz (1200 rpm) with no load, 0 V (0 Nm), and a rotational speed of 30 Hz (1800 rpm) with a load of 2 V (7.32 Nm). The five bearing operating conditions were normal (N0), ball fault (BF), inner-ring fault (IF), outer-ring fault (OF), and combined inner- and outer-ring fault (CF). The bearing dataset was obtained through data collection. During the experiment, eight sensors monitored the experimental platform. The installation location and function of each sensor are shown in Table 3.

In practical applications, the amount of data related to mechanical failures is much less than the normal data. To reflect the actual situations, for normal conditions, the training set, validation set and test set are much more than other failure conditions. The number of training, validation, and test samples for bearing failure types and various working states in the power transmission simulation experimental platform is shown in Table 4. The number of training samples with normal conditions is much larger than that of other failure types, with a proportion of 71.4%.

In the initial stage of the experiment, a comparative analysis was conducted to examine the impact of different hyperparameters on the experimental results. The experimental conditions were set as follows: the batch size is 64, the optimizer is ‘Adam’, the learning rate is 0.01, the effective number of training epochs is 100 and the stopping criteria is when the loss does not decrease for 10 epochs. The experimental results are the average over three random initializations.

### 3.2. Results and Discussion

#### 3.2.1. Diagnostic Results

**Figure 7:**
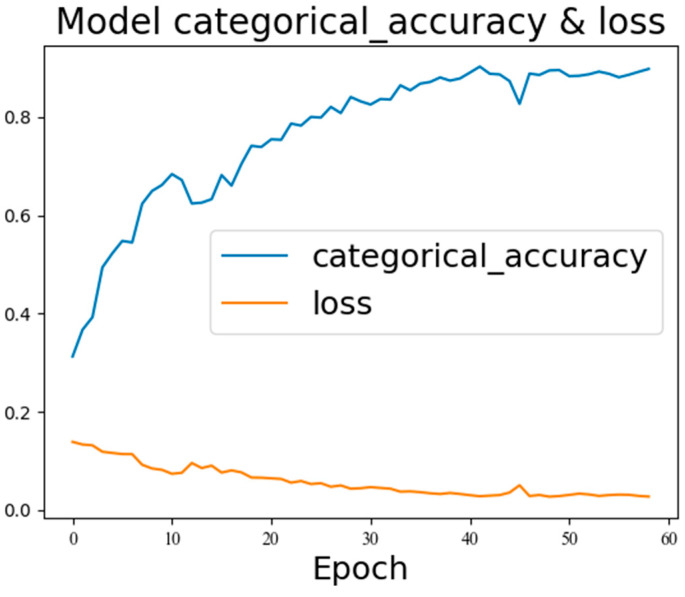
The diagnostic results of the SCGAT model using the superior-sensor group.

After training the model with the bearing dataset, the sensitivity indices of each sensor were determined. Through sensitivity analysis, sensors with sensitivity indices lower than the average value were selected. Then, sensors with strong correlations with each other were screened out through correlation analysis; the sensor with a lower sensitivity index was selected. The selected sensor group was used as the input for fault diagnosis. If the diagnostic results were satisfactory, the selected sensor group would be regarded as the optimal sensor group. Otherwise, the selection range of sensors would be expanded, and the above steps would be repeated until the desired results were obtained. Under the condition of a ratio of normal samples to faulty samples of 10:1, the diagnostic results showed an accuracy rate of 98.01% and a loss value of 0.0059, as shown in Figure 7. This verified that the proposed SCGAT model in this paper can improve the classification accuracy and stability in the case of an imbalanced dataset.

**Figure 8:**
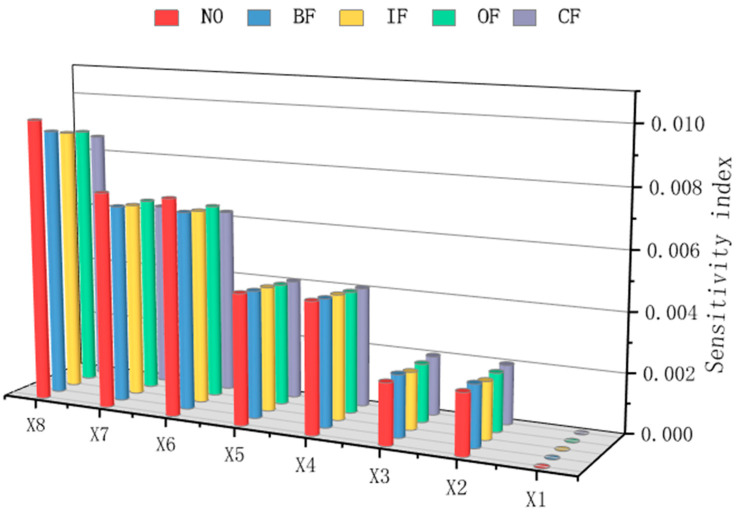
Total sensitivity index of each sensor for each type of fault.

Different sensor groups are selected for fault diagnosis based on different fault types. By integrating various sensor groups of all fault types, the optimal sensor group is obtained through analysis and comparison. Conducting a sensitivity analysis for five types of faults once cannot determine which sensors are more sensitive to each type of fault. Therefore, sensitivity analyses are conducted for each of the five types of faults separately, and the total sensitivity index and average sensitivity index of each sensor under various faults are obtained. The results are shown in Figure 8.

Remove each type of low-sensitivity sensor and form a low-sensitivity-sensor group. Since the sensitivity index values of the sensors are all relatively low, sensors with sensitivity indices lower than the average value are selected as the corresponding state’s low-sensitivity sensors and sorted according to the average sensitivity index. The average sensitivity index is the average of the total sensitivity indices corresponding to the five states. In this case, the sensors selected for each fault condition are X1, X2, X3, and X4. Place the low-sensitivity sensor group into the SCGAT model for re-training and testing. The diagnostic accuracy rate is 89.6%. The result is within expectations. The possible reasons for this phenomenon are the significant reduction in sensors and the fact that there are strongly coherent sensors among the sensors. If sensors have a high correlation with each other, they may interfere with each other during diagnosis and identification.

**Figure 9:**
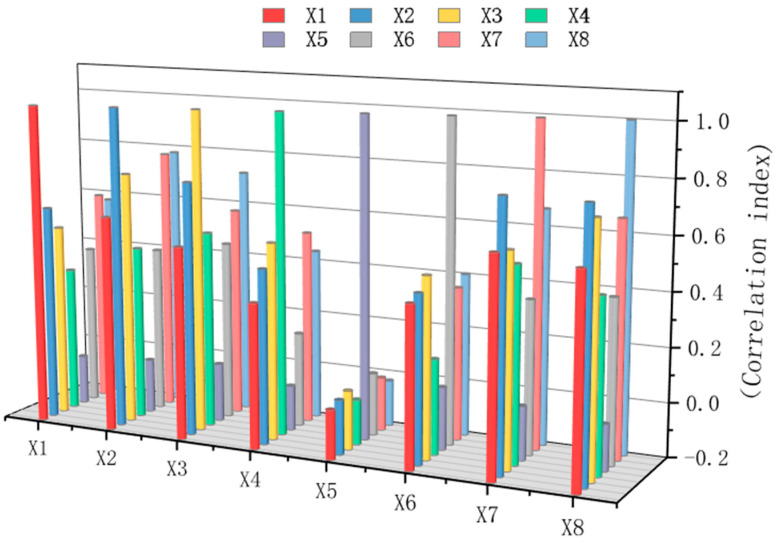
Correlation between each pair of sensors.

Conduct pairwise correlation analysis for all sensors according to different fault types and remove redundant sensors. The correlation coefficient between each sensor is shown in Figure 9. The correlation coefficients of 0.6 and above include X1 and X2, X2 and X7, X8, and X3 and X8. Clean and merge the sensors with correlation coefficients of 0.6 and above, and remove the sensors with a higher average sensitivity index. To ensure that the number of sensors is not too small, observe from the correlation graph and find that X4 and X5 have correlations with multiple sensors, which may contain more information, and their average sensitivity index is also low, so they are selected as reserved sensors. The reserved sensors and the filtered sensitive sensors form a sensitive-sensor supplementary set, and they are re-trained and tested in the SCGAT model. Under the condition of a normal sample-to-fault sample ratio of 10:1, the diagnostic result shows an accuracy rate of 98.01%.

**Figure 10:**
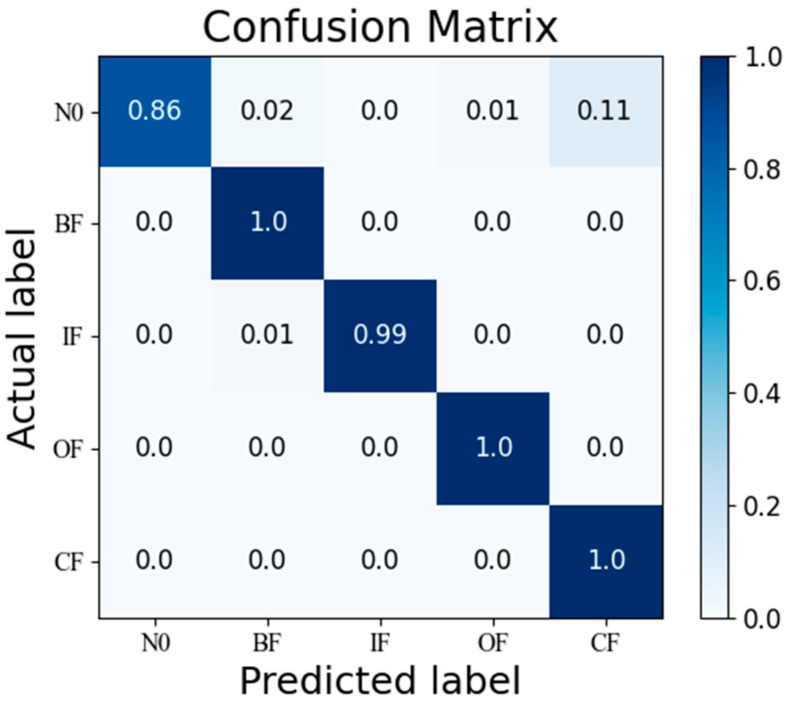
Confusion matrix obtained by using the SCGAT method.

**Figure 11:**
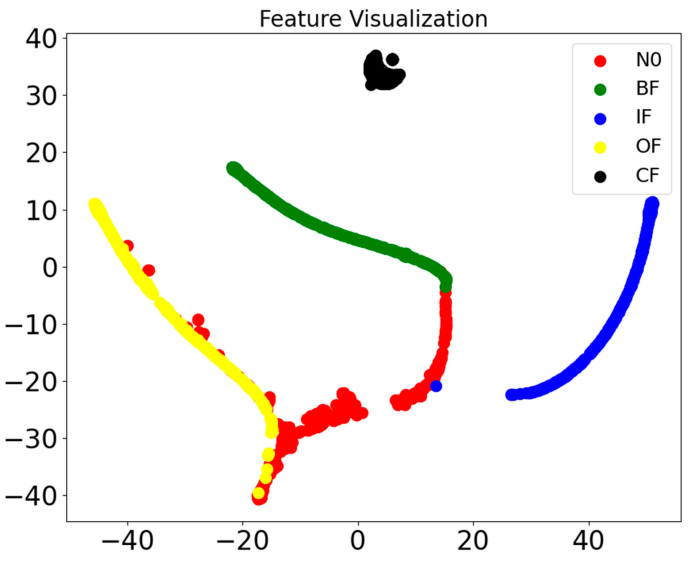
A visualized graph of features obtained by using the SCGAT method.

**Figure 12:**
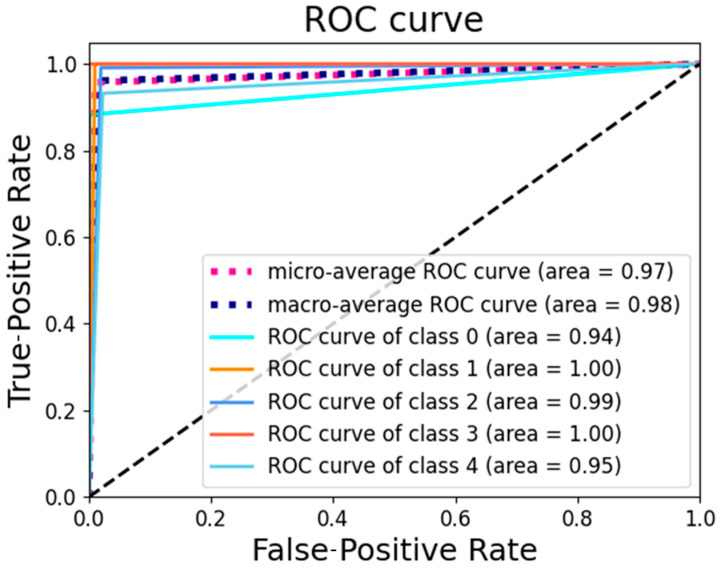
The ROC curve obtained by using the SCGAT method.

The fault diagnosis results from the additional set of low-sensitivity sensors met the expectations. The test results calculated using the SCGAT method are shown in Table 5, the confusion matrix calculated using the SCGAT method is shown in Figure 10, the feature visualization is shown in Figure 11, and the ROC curve is shown in Figure 12. If the expected result was not achieved, there is still potential for further improvement. For these five types of faults, sensors with the lowest indices from the corresponding medium-sensitivity sensors can be selected. If such sensors are already included in the low-sensitivity parameter-cleaning supplementary set or the low-sensitivity parameter set, one more sensor can be added. If there are still sensors in the selected sensors that have a strong correlation with the previous sensor group, they should be eliminated. The remaining ones can be used as a secondary supplementary set to supplement the optimal sensor group. The obtained optimal-sensor-group data can be used as the input for the diagnostic model. The data from the optimal sensor group can be used to re-train and optimize the SCGAT model and test its diagnostic performance until the diagnostic accuracy requirement is met.

#### 3.2.2. Ablation Experiment

**Figure 13:**
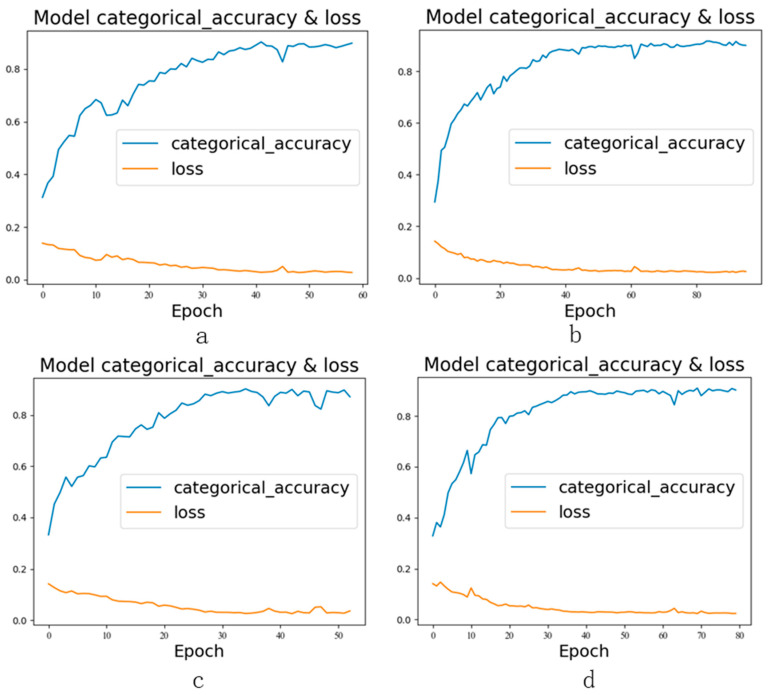
Accuracy and loss curves of different experimental groups in the ablation experiment: (**a**) Original sensor group; (**b**) sensor group selected through sensitivity analysis without correlation analysis; (**c**) sensor group selected through no sensitivity analysis but with correlation analysis; (**d**) sensor group selected after both sensitivity analysis and correlation analysis.

After conducting sensitivity analysis and correlation analysis on the sensors, the following four groups of sensors were tested to verify the effectiveness of the sensor selection algorithm. The data from each group of sensors were used as the input of the SCGAT model, and the test results are shown in Table 6 and Figure 13: (1) The original sensor group X1 to X8. (2) The sensor group selected based on sensitivity analysis without correlation analysis, according to the total sensitivity index of each sensor under each type of fault in Figure 8; sensors X1, X2, X3, X4, X5, X6, and X7 were selected. (3) The sensor group selected based on correlation analysis without sensitivity analysis; the correlations between sensors are shown in Figure 9; the sensor group was X2, X3, X4, X5, X6, and X8. (4) The sensor group selected through sensitivity analysis and correlation analysis—X1 to X4.

From the test results, the test accuracy of group (1) is 98.01%, which is the highest among the comparison groups. The possible reason for this is that the bearing dataset has few sensors, which leads to information loss after sensor selection. In the other three groups, the accuracy of group (4) is 0.9361, which is the highest among these three groups, verifying the effectiveness of the sensor selection algorithm. The accuracy rate of group (2) is higher than that of group (3), indicating that the sensor group selected through sensitivity analysis carries more effective information than the sensor group selected through correlation analysis. Although the accuracy of group (4) was lower than that of group (1), the test results met the expectation.

#### 3.2.3. The Impact of Imbalanced Datasets on Diagnostic Results

The proposed SCGAT method is mainly aimed at the fault diagnosis task with an imbalanced dataset. To evaluate its performance, normal samples and fault samples are divided into different ratios, such as 5:1, 10:1, 30:1, 50:1, 70:1 and 100:1. Take this as the input of the SCGAT method to conduct fault diagnosis. The results are shown in Table 7.

From the results, when the ratio of normal samples to fault samples is 5:1, the accuracy rate is the highest, reaching 98.94%. As the sample ratio gap increases, the accuracy rate gradually decreases. When the sample ratio is 100:1, the accuracy rate is the lowest. However, when the sample ratio is within 50:1, the test accuracy rate does not significantly decrease. In order to observe how the rare classes behave under different imbalance ratios, the precision, recall rate and F1-score of outer-ring fault (OF) samples are shown in Table 8. From the results, when the ratio of normal samples to fault samples is 50:1 and 70:1, the precision is 0.83 and the recall rate is 0.88 respectively; in addition to this, all the other results have met the expectations. In summary, SCGAT shows a high accuracy rate and can be used for bearing fault diagnosis tasks under imbalanced-dataset conditions.

#### 3.2.4. Comparison with Other Methods

To evaluate the performance of SCGAT, a comparison was made with other methods, including SAE-DNN, GRU, BiGRU, LFGRU, and CNN, as well as MobileNet-V2, ShuffleNet-V2, etc. [44,45,46]. In this experiment, 1024 sampling points were selected as one training sample. To reflect the fault conditions in practical applications, for normal cases, the training set took 700 samples, while the validation set and test set each took 150 samples. For other faults, 70 samples of each type were taken as the training set, and 15 samples each were taken as the validation set and test set. The SCGAT model adopted set the output layer to five neurons, corresponding to five different operating states’ labels. Finally, the fault diagnosis accuracy rates of all models on the dynamic-simulation experimental platform of the power transmission system were obtained, and the comparison results are shown in Table 9. 

From the results, compared with other machine learning methods, the SCGAT network model has the highest fault diagnosis accuracy rate and achieves the best performance. Moreover, compared with the convolutional neural network model with better performance, the SCGAT network model can improve the classification accuracy of fault diagnosis. Specifically, under the experimental condition of gear which the rotational speed is 1200 rpm (20 Hz) and without load (0 V), it has higher accuracy compared to MobileNet-V2. It was proved that the SCGAT method also has advantages in accuracy, in addition to solving fault diagnosis under imbalanced datasets.

The experimental results show that the SCGAT network model can learn useful features from multiple sensor information, which is conducive to distinguishing fault types accurately and thereby improving the classification accuracy of the fault diagnosis system. The SCGAT model still shows satisfactory results on the limited available dataset, with an average accuracy rate of up to 98.9%. The relevant information in the constructed topology graph greatly improves the accuracy and stability of the model, so the proposed SCGAT model is very suitable for bearing fault diagnosis under imbalanced-dataset conditions.

## 4. Conclusions

The amount of data related to mechanical failures is much less than the normal data in many industrial scenarios, resulting in a diagnostic task with an imbalanced dataset. The SCGAT is a fault diagnosis method proposed to diagnose the faults in imbalanced datasets. Firstly, a graph attention convolutional neural network model, which integrates the advantages of GAT and CNN, is constructed and pre-trained. Secondly, selecting the superior-sensor group through sensitivity analysis and correlation analysis can decrease the computational load and lower the cost of sensors, and can also can enhance the efficiency of feature extraction by decreasing the unwanted signal. Finally, the data of the selected sensor group, as the input of the model, was used to train the graph attention convolutional neural network model. The trained model is utilized for feature learning and fault diagnosis.

Diagnostic results show that the SCGAT method can diagnose faults in an imbalanced dataset effectively. In an ablation experiment, conducting sensitivity analysis and correlation analysis on the sensors, the four groups of sensors were tested to verify the effectiveness of the sensor selection algorithm. The experiment, in which normal samples and fault samples are divided into different ratios, such as 5:1, 10:1, 30:1, 50:1, 70:1 and 100:1, verified that this method can diagnose the fault accurately. The comparative studies show that it also has advantages in accuracy. The proposed SCGAT method has specific industrial application value.

However, several aspects of the paper warrant further polishing and improvement. Firstly, the approach is dependent on a single test bench and a single type of machinery, and it does not generalize to other rotating systems or to datasets with even more extreme imbalance. Secondly, it does not utilize or contrast explicit imbalance-sensitive approaches like cost-sensitive loss, focal loss, or re-sampling. Finally, the sensitivities and the cut-offs of the correlations are unjustified both in theory and in empirical studies; these need to be adjusted based on the fault diagnosis results. A potential improvement for this is to find a threshold value that is applicable to most scenarios, or adjust it dynamically in a simple way to adapt to most scenarios.

## Figures and Tables

**Table 1:** Classification of sensitivity levels.

$\mathrm{Sensitivity}\;\mathrm{Index}\;S_{T}$	Sensitivity Level
$0.00\leq |S_{T}|<0.05$	Inefficient
$0.05\leq |S_{T}|<0.20$	Moderately sensitive
$0.20\leq |S_{T}|<1.00$	Sensitive
$1.00\leq |S_{T}|$	Highly sensitive

**Table 2:** Relationship between correlation coefficient values and correlation strength.

Correlation Coefficient	Correlation Strength
0.8–1.0	Very strong correlation
0.6–0.8	Strong correlation
0.4–0.6	Moderate correlation
0.2–0.4	Weak correlation
0.0–0.2	Extremely weak correlation or no correlation

**Table 3:** Sensor description of the power transmission simulation experimental platform.

Sensor	Installation Location	Description
X1	Motor	Motor vibration
X2	Planetary gearbox	Vibration in the x direction
X3	Planetary gearbox	Vibration in the y direction
X4	Planetary gearbox	Vibration in the z direction
X5	Motor	Motor torque
X6	Parallel gearbox	Vibration in the x direction
X7	Parallel gearbox	Vibration in the y direction
X8	Parallel gearbox	Vibration in the z direction

**Table 4:** Bearing failure types, training, validation and test sample quantities.

Working Status	Total	Training	Validation	Testing	Description
N0	1000	700	150	150	Healthy
BF	100	70	15	15	Roller has cracks
IF	100	70	15	15	Inner ring has cracks
OF	100	70	15	15	Outer ring has cracks
CF	100	70	15	15	Both inner and outer rings have cracks

**Table 5:** Test results calculated using the SCGAT method.

No.	Working Status	Precision	Recall Rate	F1-Score
1	N0	0.94	0.86	0.96
2	BF	0.99	1.00	0.99
3	IF	1.00	0.99	0.99
4	OF	0.99	1.00	0.95
5	CF	1.00	1.00	1.00

**Table 6:** Test results of ablation experiment.

No.	Sensor Group	Accuracy Rate	Precision	Recall Rate	F1-Score
1	Sensor group X1 to X8 (8 sensors)	0.9801	0.9828	0.9801	0.9809
2	Sensor group X1 to X7 (7 sensors)	0.9163	0.9280	0.9163	0.9203
3	Sensor group X2 to X6, X8 (6 sensors)	0.8051	0.7091	0.7954	0.8051
4	Sensor group X1 to X4 (4 sensors)	0.9361	0.94	0.94	0.94

**Table 7:** Test results of normal samples and fault samples at different proportions.

No.	Ratio of Normal Samples to Faulty Samples	Testing Accuracy	Precision	Recall Rate	F1-Score	Loss
1	5:1	0.9894	0.9925	0.9894	0.9912	0.004
2	10:1	0.9801	0.9828	0.9801	0.9809	0.0059
3	30:1	0.9442	0.9389	0.9442	0.9519	0.0226
4	50:1	0.8913	0.9088	0.8913	0.8690	0.0422
5	70:1	0.8233	0.7153	0.8233	0.7393	0.0690
6	100:1	0.8210	0.9070	0.8210	0.9396	0.0712

**Table 8:** Test results of outer-ring fault (OF) samples at different proportions.

No.	Ratio of Normal Samples to Faulty Samples	Precision	Recall Rate	F1-Score
1	5:1	1.00	1.00	1.00
2	10:1	0.99	1.00	0.99
3	30:1	1.00	1.00	1.00
4	50:1	0.83	1.00	0.90
5	70:1	1.00	0.88	0.94
6	100:1	1.00	0.97	0.99

**Table 9:** Testing accuracies of different methods.

No.	Classifiers	Testing Accuracy (%)
1	SAE-DNN	87.5
2	GRU	91.2
3	BiGRU	93.0
4	LFGRU	93.2
5	CNN	97.3
6	ShuffleNet-V2	98.2
7	MobileNet-V2	98.6
8	SCGAT	98.9

## Data Availability

The datasets analyzed during the current study are available in the GitHub repository, https://github.com/cathysiyu/Mechanical-datasets (accessed on 30 May 2022).

## Abbreviations

The following abbreviations are used in this manuscript:

DBN	Deep Belief Network
CNN	Convolutional Neural Network
CatBoost	Categorical Boosting
DPGCN	Graph Convolutional Network based on the Distance and Probability topological graphs
1DCNN	1D Convolutional Neural Network
GraphSAGE	Graph Sample and Aggregate
SCGAT	Graph Attention Convolutional Neural Network model based on Sensitivity analysis and Correlation analysis
BF	Ball bearing Fault
IF	Inner-ring Fault
OF	Outer-ring Fault
CF	Inner- and Outer-ring Fault
SAE-DNN	Sparse Autoencoder Deep Neural Network
GRU	Gated Recurrent Unit
BiGRU	Bidirectional Gated Recurrent Unit
LFGRU	Linear-Feedback Gated Recurrent Unit
